# Predicting Electronic Health Record Usability: Scoping Review of Adoption Models, Metrics, and Future Directions

**DOI:** 10.2196/86076

**Published:** 2026-04-08

**Authors:** Dillon Chrimes, Alex Thomo, Mu-Hsing (Alex) Kuo, Elizabeth M Borycki, Andre Kushniruk

**Affiliations:** 1 School of Health Information Science, Faculty of Health, University of Victoria, PO Box 1700 STN CSC, Victoria, BC, V8W 2Y2, Canada, 1 250-857-2404; 2 Computer Science, Faculty of Engineering & Computer Science, University of Victoria, Victoria, BC, Canada

**Keywords:** artificial intelligence, electronic health records, perceived usefulness, predictive analytics, technology acceptance model, usability

## Abstract

**Background:**

Electronic health records (EHRs) play an essential role in modern health care, enabling data sharing and improving patient safety; however, even though vendors must adhere to International Organization for Standardization–related usability standards for EHR certification, persistent usability issues continue to undermine efficiency, contribute to clinician burden, and increase the risk of preventable errors.

**Objective:**

This scoping review synthesizes existing research on EHR adoption and usability, emphasizing theoretical models, measurement approaches, factors, and analytic methods used to assess or predict usability. We identify gaps and opportunities for integrating predictive analytics and artificial intelligence (AI) to advance research and improve the usability of EHRs.

**Methods:**

Following Joanna Briggs Institute and PRISMA-ScR (Preferred Reporting Items for Systematic Reviews and Meta-Analyses extension for Scoping Reviews) guidelines, we systematically searched MEDLINE, Web of Science, IEEE Xplore, and Scopus library databases for studies published between January 1, 2009, and April 9, 2025. Inclusion criteria focused on empirical research using predictive methods or models related to EHR usability. Data were charted and synthesized thematically.

**Results:**

Of the 2323 screened papers, 47 studies met inclusion criteria. Most research examined or predicted EHR adoption (not usability) using dominant frameworks, such as the technology acceptance model, unified theory of acceptance and use of technology, and the information system success model, which comprised usability. Factors related to usability—particularly perceived usefulness, perceived ease of use, effort expectancy, and facilitating conditions via the EHR adoption models—appeared frequently. Regression-based methods and structural equation modeling were the most common analytic techniques. No studies applied predictive modeling or AI to predict EHR usability.

**Conclusions:**

The focus of this study on the prediction of EHR usability and adoption for the past 15 years is distinctive in the literature. It extends prior usability reviews (mostly focusing on adoption, not prediction of usability). Predictive modeling for EHR usability remains underdeveloped throughout 2009 to 2025. Dominant frameworks in the EHR literature continue to prioritize predicting adoption over operational usability. These models rely heavily on self-reported, cross-sectional measures captured at a single postimplementation time point, embedding systematic bias and obscuring longitudinal usability dynamics. Despite the application of increasingly sophisticated predictive techniques—primarily variants of regression and structural equation modeling—usability has remained analytically subordinate to adoption and acceptance constructs for more than 15 years. As a result, widely used models, such as the technology acceptance model and unified theory of acceptance and use of technology, position usability merely as an antecedent to intention or use, rather than as an independent, system-level property that can be empirically measured, modeled, and predicted. Therefore, there is substantial opportunity to integrate predictive analytics, AI, and longitudinal usability measures to build dynamic models.

## Introduction

Electronic health records (EHRs) are central to modern health care delivery, enabling real-time access to patient information, supporting continuity of care, and enhancing clinical decision-making. However, persistent usability challenges continue to increase cognitive workload, reduce user satisfaction (US), and contribute to patient safety risks [1-5]. Usability can be defined as the extent to which a system can be used efficiently, effectively, and satisfactorily by specified users in a specified context (International Organization for Standardization [ISO] 9241‐11) [6]. In contrast, adoption refers to the extent to which users accept and implement technology [7]. Usability can directly influence the uptake of the system; however, it is not the same as adoption.

Studies show that inadequate usability contributes to data entry errors, inefficient workflows, increased cognitive burden, and user frustration, thereby limiting the potential benefits of EHRs [8,9]. While EHR vendors adhere to certification processes, these often fail to capture human factors that shape system utility and usefulness [10,11]. Reliable measures of the usability of EHRs—particularly those that can predict performance before and after go-live—remain scarce, and even fewer studies have examined usability over extended durations post implementation of ≥1 year [12].

Over the past 2 decades, theoretical frameworks, such as technology acceptance model (TAM), unified theory of acceptance and use of technology (UTAUT), and the DeLone and McLean (D&M) information system success (ISS) model, have been widely applied to understand EHR adoption and acceptance. Core determinants in the constructs of these theoretical frameworks, such as perceived usefulness (PU) and perceived ease of use (PEOU), consistently predict user intentions and behaviors [13,14]. However, these adoption models often rely on cross-sectional surveys to collect data to run the model and do not comprise any ISO or industry standards, offering limited insight into how the EHR system performs, as usability evolves over time. Hill et al [7] concluded that despite the widespread adoption of EHR systems, a very small number of research papers analyzed the use, impact, and weaknesses of existing search functions for clinical tasks. Moreover, EHR usability changes dynamically with user experience and across the software development life cycle [15,16], which EHR adoption frameworks do not capture.

Many EHR interfaces remain cluttered, nonintuitive, and difficult to navigate, requiring ongoing training [1]. Such design limitations increase cognitive workload, prolong documentation time, and contribute to alert fatigue and workflow interruptions [17-19]. Physicians spend nearly half of their workday interacting with EHRs [20]. Alert fatigue, task switching, and interoperability failures further reduce usability standards and compromise care coordination [21,22].

A range of methods has been employed to assess usability. Heuristic evaluations allow for comparisons of EHRs against usability principles [23,24], while clinician-based testing highlights workflow inefficiencies and inadequate training [25]. Surveys reveal widespread dissatisfaction, with 70% of physicians citing usability concerns [26]. PU indicators include improved performance, productivity, and effectiveness [27], whereas PEOU indicators include learnability, clarity, flexibility, and ease of becoming skillful [28]. The D&M ISS model extends these perspectives with constructs such as system quality (SQ) and information quality (IQ) [29-31]. However, these models typically lack task-level analyses, despite evidence that inefficient task flows waste time, increase errors, and reduce physician satisfaction, altering the behavior, intent, and acceptance of adopting the EHR workflow [32-35].

Audit logs and system metadata provide opportunities to quantify usability through measures such as task completion time, error rates, system usability scale (SUS) scores, National Aeronautics and Space Administration-Task Load Index (NASA-TLX) scores for cognitive load, clickstream and keystroke logs, US surveys, and eye-tracking data [24,36,37]. Yet these metrics are not captured by EHR adoption models and are rarely used to predict specific usability problems or outcomes.

Emerging approaches in data mining, human-computer interaction (HCI), and artificial intelligence (AI) offer new opportunities for predictive modeling of usability. Techniques such as logistic regression, decision trees, random forests, gradient boosting (eg, XGBoost), support vector machines, and neural networks—including recurrent neural networks, convolutional neural networks, and long short-term memory networks—have been applied to analyze system logs, classify sessions, and model usability [38-40]. Predictors such as interaction complexity (eg, clicks per task), alert fatigue, workload peaks, demographic variables (eg, age and digital literacy), and prior EHR training have also been identified [19,21,25,31,37]. Integrating such factors into dynamic feedback loops could enable continuous monitoring and improvement for safety-enhanced design with vendors and end users in clinical settings. However, the extent to which these approaches have been systematically applied in EHR usability research remains to be fully studied.

Given the large datasets (eg, Office of the Coordinators Certified Health Product List dataset of usability testing results for EHR certification [41] that had been collecting data on EHR usability from 2014 until the present), this knowledge could be useful for predicting and anticipating potential usability problems with specific EHRs. Additionally, the data could be mined and used for supporting, selecting, and procuring usable EHRs for health care organizations that match their contexts, as well as for predicting and anticipating usability issues that may occur over time.

To address these gaps, we conducted a scoping review to examine any existing research that predicts the usability of EHRs with special emphasis on postimplementation. The main research objective was to examine and identify gaps in the literature on the prediction of EHR usability that could follow how theoretical frameworks, usability metrics, and analytic methods have been applied. By doing so, we aimed to critique existing models and highlight opportunities for predictive factors in EHR usability research and any further development of dynamic approaches to predict usability. These insights into the usability of EHRs over time and across systems are of value to health care organizations, system designers, EHR vendors, government certification bodies, and clinicians.

## Methods

### Design and Framework

#### Methodology and Data

This scoping review began on February 10, 2023. Our review followed the Joanna Briggs Institute (JBI) scoping review methodology and adhered to the PRISMA-ScR (Preferred Reporting Items for Systematic Reviews and Meta-Analyses extension for Scoping Reviews) reporting guidelines (Checklist 1) [42]. The sequential steps defined by Arksey and O’Malley [43] were applied: (1) identifying the research question, (2) identifying relevant studies, (3) selecting studies for inclusion, (4) charting the data, and (5) collating, summarizing, and reporting the results.

Four electronic databases were searched: MEDLINE (Ovid), Web of Science, IEEE Xplore, and Scopus. The MEDLINE database was selected because it comprises operational research on EHRs in clinical settings, nursing, health care, and related fields from 1946 to the present. Similarly, the Web of Science database was selected because it contains research on EHR usability in clinical settings, HCI journals, implementation science, and human factors research on EHRs across sciences, social sciences, arts, and humanities. The IEEE Xplore database was selected because it contains papers about HCI with EHRs and technical considerations of implemented EHRs related to usability across computer science, engineering, and electronics. Finally, the Scopus database was selected because it contains human factor research across scientific and technical fields (health, physical, and social science) plus arts and humanities.

#### Step 1: Identifying the Research Question

The research questions were as follows:

1. What is the state of the literature relevant to the prediction of EHR usability?
2. What studies have been conducted that predict or model EHR usability?
3. What factors are used to predict EHR usability?
4. Which methodologies have been used to evaluate or predict EHR usability?

#### Step 2: Protocol Registration, Screening Reliability, and Data Charting Procedures

This scoping review was conducted in accordance with the JBI methodology and reported using PRISMA-ScR guidelines. A formal review protocol defining the research questions, eligibility criteria, search strategy, and data charting framework was developed a priori and finalized prior to database screening. To ensure screening reliability, 3 independent reviewers (DC, EMB, and AK) conducted both title/abstract and full-text screening, with conflicts resolved through structured consensus meetings. Interreviewer agreement was monitored throughout the screening process, and discrepancies were discussed until full agreement was achieved, thereby strengthening internal validity. The reviewers AW, EMB, AK, AT, and DC have published and worked with EHRs, and all have experience and expertise as content experts and as methodological experts.

Data charting followed a structured extraction framework that systematically captured study design, theoretical models, analytic methods, usability metrics, predictors, and outcomes. Charting was conducted iteratively and audited across multiple cycles to ensure internal consistency and completeness. Inclusion and exclusion criteria were defined a priori with a clear rationale grounded in the study objective of identifying predictive—not purely descriptive—approaches to EHR usability, thereby justifying the exclusion of studies focused solely on organizational acceptance, vendor reports, non-EHR systems, or clinical outcome prediction without a usability construct. These safeguards ensured methodological transparency, reproducibility, and commitment to JBI and PRISMA-ScR standards.

#### Step 3: Identifying Relevant Studies

Eligibility criteria were defined using inclusion and exclusion rules that determine whether a study was relevant to the research question (Table 1). Inclusion and exclusion criteria were established before the first screening was carried out. The criteria specified study characteristics, such as publication timeframe, focus on usability, predictive methods, and EHR/electronic medical record (EMR) context for inclusion.

**Table 1.** Inclusion and exclusion criteria in the search strategy.

Domain	Inclusion criteria	Exclusion criteria
Modeling frameworks	Adoption- or sociotechnical-related factors linked to usability Regression analyses or modeling approaches aimed at predicting usability Analytic approaches and measurable usability in EHR^a^ adoption frameworks	Papers describing organizational-level acceptance only Papers linking usability to safety only Health information technology (HIT) only
Electronic records	EMR^b^ and EHR, or broader health information system contexts EMR and EHR adoption	Dental systems, medication systems, and pharmacy Personal health record General information systems, eye care, and radiology Surveillance Laboratory information systems
EHR adoption and usability	Technology acceptance or sociotechnical models relevant to usability User-centered design, tasks, social-technical User acceptance, patient safety User satisfaction, human-computer interaction Safety-enhanced design Cognitive task load	Usability survey only Usability of other systems User experience only
Informatics and data used in the model	Usability data modeled User experience data modeled EHR adoption	EHR data, Public Health Informatics Patient data and EHR phenotyping Medical conditions Health information exchange Clinical data
Artificial intelligence	Data mining, regression, confirmatory analysis, hierarchical regression, and exploratory factor analysis Text mining Algorithm Machine learning and deep learning	Pattern recognition and large language models
Context	Prediction or analysis EHR or EMR	Clinical or solely clinical context
Time frame	From January 2009 to April 9, 2025	Published before 2009
Study design	Usability research studies Qualitative and quantitative analyses Cognitive load with the EHR usability model Usability data analysis of EHRs	No literature reviews, commentary, or opinion Papers without full text EHR data analysis for medical outcomes No technical reports No position or opinion viewpoints No vendor-based reports

a EHRs: electronic health records.

b EMR: electronic medical record.

These inclusion criteria were defined to capture studies using quantitative, qualitative, or mixed methods approach that assessed, modeled, or predicted aspects of EHR usability, EHR adoption, or sociotechnical factors primarily. The exclusion criteria removed research papers that were not related to EHRs, studies focused on usability but not prediction, and excluded literature reviews, opinion papers, or technical reports. All papers included were in English only.

### Search Strategy

A comprehensive search was conducted across 4 databases—MEDLINE (Ovid), Web of Science, IEEE Xplore, and Scopus—to identify studies evaluating or predicting EHR usability. All searches were limited to English-language publications from January 1, 2009, to April 9, 2025. Keyword strategies incorporated terms related to usability, prediction or analytics, and EHRs.

The search strategy included combinations of controlled vocabulary terms and keywords related to usability (“usability,” “user experience,” “human factors,” “human-computer interaction”), prediction (“predict*,” “regression,” “data mining,” “machine learning”), and EHR (“EHR,” “electronic health record,” “EMR”).

Search strategies for each of the 4 databases were as follows:

1. IEEE database ((((usability OR user OR “human factors” OR “human-computer interaction”)))) AND (("predict*" OR “classif*” OR forecasting OR “data mining” OR “text mining” OR “regression*” OR “confirmatory factor analy*”) AND (EHR OR “electronic health record*” OR EMR OR “electronic medical record*”)).
2. Web of Science (usability or user or “human factors” or “human-computer interaction”) AND (predict* or classif* or forecasting or “data mining” or “text mining” or “regression*” or “confirmatory factor analy*”) AND (EHR or “electronic health record*” or EMR or “electronic medical record*”).
3. MEDLINE (Ovid) ((usability or user or “human factors” or “human-computer interaction”)).tw,kf. AND ((“predict*” OR “classif*” OR “data mining” OR “regression*” OR “confirmatory factor analy*” OR algorithm*)).tw,kf. AND (EHR or “electronic health record*” or EMR or “electronic medical record*”).tw,kf.
4. Scopus (TITLE-ABS ((usability OR user OR “human factors” OR “human-computer interaction”)) AND TITLE-ABS ((“predict*” OR “classif*” OR “data mining” OR “regression*” OR “confirmatory factor analy*”)) AND TITLE-ABS (ehr OR “electronic health record*” OR emr OR “electronic medical record*”)) AND (LIMIT-TO (LANGUAGE, “English”)) AND (LIMIT-TO (SRCTYPE, “j”) OR LIMIT-TO (SRCTYPE , “p”)).

### Screening, Data Extraction, and Analysis

#### Step 4: Study Selection

Title and abstract screening were completed by 3 reviewers, namely EMB, AW, and DC. Each reviewer independently screened the titles and abstracts of papers, with conflicts resolved by consensus during regular review meetings via Covidence. Title and abstract screening established the study section as a quick review of papers that the reviewers deemed included or excluded based on the inclusion and exclusion criteria. For papers that met the eligibility criteria, the papers were analyzed using a conventional content analysis approach to review the papers, chart the data, and identify themes.

For papers that did not meet the criteria, the reviewers noted this. Once the first screening was completed, the second full-text screening was then conducted to determine final eligibility. Studies were excluded during screening if they did not address usability or EHR-related usability via EHR adoption models; did not include predictive or analytic methods; did not directly examine EMR/EHR usability; lacked empirical methods or analytic rigor, or had the wrong study design.

The screening workflow ensured that only studies with explicit relevance to usability measurement, modeling, or prediction were retained.

Data extraction was carried out using a structured template. Extracted variables included study design, country and year, models or frameworks used, adoption determinants, usability-related metrics or instruments, analytic methods (eg, regression, SEM), main predictors examined, and key findings. Extraction fields were defined a priori to ensure consistency and comprehensiveness across studies.

#### Steps 5 and 6: Charting, Collating, and Reporting with AI Method Utilization

Study characteristics were manually charted by the research team. These characteristics included country of study, publication year, keywords, study design, analytic method, and predictive or explanatory techniques.

Extracted variables and categorizations were cross-checked using ChatGPT (GPT-5) to identify inconsistencies, ensure terminology alignment, and cross-check study characteristics. It should be noted that GPT-5 was used after manually updating the critique of the 47 journal papers (2009-2025) in Table S1 in Multimedia Appendix 1. It did not reproduce that table. For each of the papers selected for full review, GPT-5 was prompted with the instruction “summarize the study design, models, factors, significant findings and overall critique” to cross-check the content of the table’s columns of models, factors, and critique. The response from GPT was mostly consistent, with only a few discrepancies found between GPT-5 and the human researcher. Any discrepancies were manually reviewed and updated thereafter. GPT-5 was only used for checking human processes in the review and critique of the papers found. GPT-5 was also used to obtain frequency counts for categories of predictive techniques in the papers. This was also cross-checked with frequency counting by the human researcher, with any discrepancies in the categories and the counts resolved through further analysis by the human researcher.

### Ethical Considerations

This study was reviewed by the University of Victoria Human Research Ethics Board (UVic HREB) Chair and determined to be exempt from formal human research ethics review because the research does not involve human participants and is limited to the analysis of existing, publicly available documents and/or datasets. The determination was made in accordance with the Tri-Council Policy Statement: Ethical Conduct for Research Involving Humans (TCPS2 2022) and the University of Victoria Board of Governors Research Policy RH8100 and University of Victoria Board of Governors Regulations for Research Involving Humans RH8105. The UVic Human Research Ethics Chair concluded that the activities described in this study fall outside the scope of research requiring institutional ethics review because they do not involve human participants, human biological materials, or identifiable private information. The work is limited to the analysis of publicly available documentation and datasets.

## Results

### Study Selection and Characteristics

**Figure 1.**
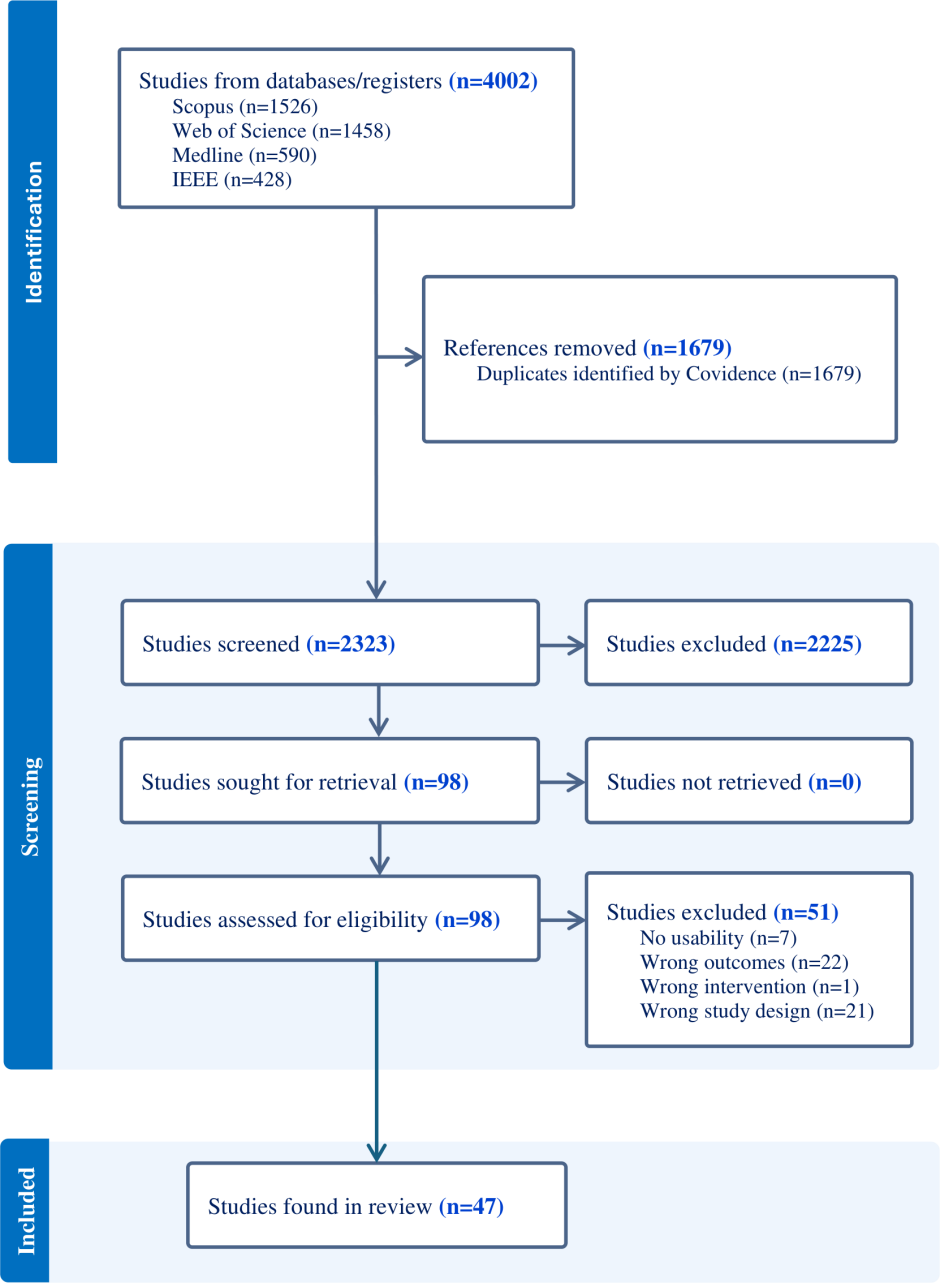
PRISMA (Preferred Reporting Items for Systematic Reviews and Meta-Analyses) flow diagram.

The included studies were drawn from 4 major databases: Scopus (n=1526), Web of Science (n=1458), MEDLINE (n=590), and IEEE (n=428). Database searches yielded 428 papers in IEEE Xplore, 1458 in Web of Science, 590 in MEDLINE (Ovid), and 1526 in Scopus. After the removal of duplicates (n=1679), a total of 2323 records underwent title and abstract review in the first screening (Figure 1). A total of 98 full-text papers were assessed during the full-text screening. In the full paper screening, 51 records were excluded because of wrong outcomes (n=22), wrong study design (n=21), studies that did not address usability (n=7), and wrong intervention (n=1). Thus, a total of 47 studies met the full screening eligibility and were retained for analysis.

From 2323 records screened, 47 studies met the inclusion criteria. The majority were cross-sectional surveys that applied TAM, UTAUT, or ISS frameworks to evaluate EHR adoption and US. Common predictors included PU, PEOU, effort expectancy (EE), and facilitating conditions (FCs).

Cross-sectional designs were the most common (31/47), followed by survey-based studies (26/47) (Multimedia Appendix 2). Almost all studies were conducted postimplementation. Data collection methods most frequently included direct data capture (36/47) and questionnaires (31/47). Regression analysis was the most common modeling approach (26/47).

### Study Characteristics, Metrics, and Data Sources

Across the 47 included studies, EHR adoption was most commonly predicted with usability measures that were predominantly subjective, relying on surveys and self-reported satisfaction. Objective indicators—such as task completion time, error rates, and audit log data—were rarely incorporated, and no study combined task-based HCI data with predictive analytics for longitudinal monitoring.

The reliance on survey-based methods, with limited integration of task-level or objective usability metrics, was a consistent finding. Acronym use across the studies (Table S1 in Multimedia Appendix 1) reflects a synthesis of 47 studies from 26 countries (2009‐2025), largely dominated by TAM, UTAUT, and D&M ISS frameworks. Analytic approaches included regression, SEM, and in a small number of studies, machine learning or decision trees. While nearly every study examined adoption or acceptance, very few explicitly focused on prediction even with the EHR adoption constructs with significant determinants.

### Model Applications

TAM and UTAUT frameworks in many studies were evaluated using regression and SEM. Some studies used confirmatory factor analysis (CFA) and exploratory factor analysis to validate constructs. A small number integrated machine learning or neural networks with SEM, but no study applied AI to predict EHR usability.

Regression was the most commonly applied predictive analytic method, followed by SEM, which was the most frequently applied analytic methods, supplemented by ANOVA, multivariate ANOVA, CFA, and exploratory factor analysis in 13 studies [18,44-55].

### Common Determinants and Constructs

Table S1 in Multimedia Appendix 1 presents 26 reported adoption-related determinants and model constructs such as TAM, tripolar model of technology acceptance, theory of planned behavior, UTAUT, and Systems Engineering Initiative for Patient Safety (SEIPS) that had usability aspects of EHR/EMR that included (1) PU, PEOU, performance expectancy (PE), user behavior (UB), and US; (2) behavioral intention (BI), EE, FCs, and job relevance; (3) IQ, IT support, organizational culture, SQ, and service quality (SRQ); (4) broader usability frameworks and scales, such as the NASA-TLX, National Usability–Focused Health Information System Scale (NuHISS), and SUS.

### Frequency of Metrics

Metrics were grouped into nine categories: (1) attitudes and social aspects, (2) behavioral and intention-related factors, (3) IQ, (4) organizational and environmental factors, (5) PU, (6) SRQ, (7) SQ, (8) US and perceived usability, and (9) work efficiency and productivity.

FC, defined as the degree to which an individual believes that an organization and its technical infrastructure exist to support the use of the system [56], was the most frequently measured determinant construct, or predictor variable, followed by PEOU, PU, and attitude. In total, more than 50 unique usability-related metrics were reported across the 15-year span of literature.

### Temporal and Geographic Distribution

The studies spanned 16 years from 2009 to 2025, with 12 published before 2015 (Multimedia Appendix 3). Study duration was, on average, less than 1 year and ranged from 2 months to 14 years. No clear temporal trend was observed, though extending the screening into 2023 to 2025 revealed an increasing number of studies. However, databases searched in 2025 only included papers published in that year up to April 9.

Geographically, the 47 studies originated from 26 countries (Multimedia Appendix 4). The United States (10 studies) and Finland (5 studies) contributed the largest number of studies found. North American studies more often employed the D&M ISS model compared to other countries, while nursing-focused studies frequently adopted the UTAUT framework.

### Keywords and Clusters

Most frequent keywords across studies were EHR, usability, TAM, and UTAUT (Multimedia Appendix 5).

Across all studies published between 2009 and 2025, no study directly predicted EHR usability using computational or longitudinal modeling techniques. Most research examined adoption outcomes, satisfaction ratings, or intention-to-use constructs rather than usability performance or workflow-based usability metrics. Psychometric determinants (eg, PU, PEOU, EE, and FC) were frequently evaluated across adoption frameworks.

Regression and SEM techniques predominated, supporting cross-sectional inferences but offering limited value for forecasting usability over time. Although FCs often emerged as a significant predictor of intention-to-use EHRs, they were rarely examined within dynamic contexts, task-based assessments, or sequential workflows that reflect actual clinical use.

There were 95 predictive techniques of analysis across 12 technique categories found, with many studies using multiple techniques (Multimedia Appendix 6). Regression-based methods dominated (40/95) [15,17,18,27,44,45,47-49,51,55,57-73] (Multimedia Appendix 7) followed by SEM (26/95) [50-55,74-77]. Furthermore, there were several regression and SEM variants in the studies found.

Only a limited number of studies incorporated AI-related techniques, but these were used for prediction of EHR adoption and not for usability. Almarzouqi et al [78] used neural networks with SEM to predict adoption, Alsyouf and Ishak [79] used decision trees with heuristic evaluation and UTAUT, and Sachdeva et al [74] used decision trees to discover behavior patterns. Cluster analysis was used to predict general BI [79] and evaluate EHR use in primary care providers [70]. Four studies used principal component analysis for dimensionality reduction and feature extraction [44,45,70,74].

## Discussion

### Key Findings

Across 47 studies, regression and SEM dominated, indicating minimal methodological progression toward predictive, automated, or real-time usability modeling.

This scoping review found that EHR adoption models dominated studies identified using usability and prediction-related keywords. However, adoption is not synonymous with usability, despite often being treated as interchangeable in the literature. As noted by Shachak et al [80], models such as TAM and UTAUT reduce complex sociotechnical properties of health information technology to individual perceptions and intentions, privileging acceptance over system-level performance and postimplementation use. Consequently, usability was assumed through adoption intent rather than directly measured or predicted.

The predominance of EHR adoption models in the reviewed studies underscores the need to address postimplementation system success. EHRs are complex sociotechnical systems in which usability emerges from interactions among interface design, configuration, organizational context, and user roles, making post hoc evaluation insufficient for meaningful intervention.

The continued use of TAM, UTAUT, and D&M ISS models highlights an enduring emphasis on explaining acceptance and satisfaction rather than modeling or forecasting usability performance in clinical practice. Developing predictive models could shift usability research from reactive post hoc assessment to proactive identification of inefficiencies, workflow burdens, and potential contributors to clinician burnout.

This scoping review highlights that although the TAM, UTAUT, and D&M ISS provide useful foundations for understanding adoption, they offer limited ability to capture evolving usability patterns with task complexity or changes in user interaction over time. Poor usability (clunky interface and workflow disruption) hinders adoption and satisfaction, causing burnout, whereas good usability (intuitive design and efficient workflows) drives adoption and better patient outcomes, even if a system is technically adopted. Thus, an EHR can be widely adopted but still have terrible usability that leads to frustration and workarounds. Therefore, predicting usability adds value to adoption and more insight as the system becomes operationalized.

The predictive focus distinguishes this study. Zhang and Walji [12] conceptualized EHR usability as a balance between intrinsic complexity (reflecting the work domain’s inherent demands) and extrinsic difficulty (stemming from interface and workflow design). Their TURF framework—task, user, representation, and function—offers a roadmap linking usability determinants such as usefulness, satisfaction, and efficiency. Despite its potential, none of the included studies applied TURF to predictive modeling of EHR usability. EHR metadata, including audit or task logs, could serve as the foundation for predictive models of usability and clinician workload or burnout [34]. Significant predictors, such as PEOU and PU, highlight opportunities for AI-driven approaches that integrate adoption constructs with task-level metadata to advance predictive usability research.

Across the 16-year period reviewed (2009‐2025), very few studies attempted to predict EHR usability directly, regardless of model sophistication. The facilitating conditions, such as the predictor variable of EHR adoption, appeared to be significant and impacted the intent to use EHRs. The FC determinant is defined as “the degree to which an individual believes that an organization and its technical infrastructure exist to support the use of the system” [56]. This psychometric determinant was deemed to be a useful metric for predicting adoption.

FCs have a direct positive effect on the intention-to-use, but this effect often diminishes after initial system use, suggesting its relevance may shift toward sociotechnical factors affecting learnability and decision-making. That is, the FCs would have linkages to sociotechnical aspects related to learnability, which is one of the factors in usability as defined by Nielsen [81]. The implications for understanding HCI and clinical decision-making suggest that survey-based constructs may insufficiently capture deeper usability determinants rooted in workflow, cognitive processes, and task structure, as mentioned by Kushniruk et al [82]. The review showed that several countries represented in their use of EHR adoption models over the past 15 years to find success or failure in the actual or intended use of EHRs postimplementation.

None of the reviewed models predicted EHR usability. In addition, no models incorporated detailed task-level inputs (eg, time-on-task, error rates, and interaction complexity) into predictions of intended or actual system use. Instead, many studies focused on PEOU or PU as attributes of usability and not tasks. Nevertheless, these attributes are important to establish (eg, easy to learn, efficient use, easy to remember, few errors, and subjectively pleasing) as recognized core usability attributes in the concept of usability engineering in the 1990s as defined by Nielsen [81]. Therefore, this result from the literature review makes it clear that predicting EHR adoption based on BI and actual use is still under investigation, with very limited modeling related to EHR usability. Importantly, the models used in the papers did not state that categories or aspects of usability and their usefulness could be dynamically utilized postimplementation.

Logistic regression or hierarchical regression via SEM with many predictive variables and hypotheses was studied in several of the papers. However, these approaches generally reflect relationships among perceptions rather than true indicators of usability performance. Aspects of task completion or failure appear directly related to usability, yet they were rarely used to establish predictive usability metrics. Furthermore, SEM in the studies [48,50-54,59,64,75,76,78,83-86] showed complex networks of hypotheses linked through latent constructs. This highlights the need for more evidence connecting specific usability issues (eg, coded problems from video-based testing [82]) with operational real-world EHR use, which takes time, and predictive models of EHR usability could reduce the burden of large and extensive usability testing.

### Interpretation of Findings

The literature indicates an increasing sophistication in applying TAM, UTAUT, and D&M ISS constructs by researchers, often through SEM. Many predictive analytics applied within these models reflect the complexity of usability-related factors. Researchers frequently tested 10 to 15 hypotheses of TAM, UTAUT, or ISS in the analysis, demonstrating rigorous statistical approaches. However, the extensive statistical testing often highlighted variability in the acceptance or intention to use the system postimplementation rather than convergence toward models capable of predicting usability. A major trend was the use of TAM or UTAUT with SEM to evaluate multiple factors—even though many of these factors could, in principle, support predictive usability modeling, for example, by linking task-based observations with survey responses.

SEM’s prominence reflects its origins in psychology and social sciences, where latent constructs such as attitudes and intentions have long been modeled through survey-based items. The Task–Technology Fit framework has also been used in SEM to assess alignment between tasks and IT [87]. CFA was commonly employed to evaluate usability parameters within hierarchical regression models [48]. However, the reviewed studies modeled adoption, satisfaction, or BI, which are important and should be done in unison with usability testing to evaluate its efficiency with UB and acceptance.

### Global Distribution and Framework Use

The 47 studies, representing 26 countries, indicate global interest in EHR adoption research. Regional differences were minimal, although the D&M ISS model was used more frequently in North America. In 2024, Rihayha and Ismaili [52] proposed a modified human–organization–technology fit model for resource-limited settings, integrating SQ, IQ, and SRQ via SEM. Hyppönen et al [48] proposed adoption predictors, including user friendliness, perceived benefits, technical problems, and cooperation metrics, applying NuHISS in Finland. Welchen et al [88] applied NuHISS in Brazil through a star-schema confirmatory analysis. Even though NuHISS is very detailed, it lacks objective usability measures such as error rates and time-on-task. The use of NuHISS across multiple countries was a major finding, though no multicountry comparative studies were identified.

### TAM and Its Determinants

Studies have updated versions of the TAM [16,50,57,58,69,76,79,89], TAM2 [66,84], and TAM3 (a hybrid of TAM and UTAUT) [63,66,78,86]. Important TAM usability determinants included PEOU and PU [16,44,48,50,54,57,58,60,63,64,66-68,70,78,83,85,89,90]. PU and PEOU can significantly predict health care professionals’ intentions to use health information systems [13,14,58,73]. Additionally, subjective norms, relevance, and computer anxiety predict intentions beyond PU and PEOU [87,88]. One study showed that PEOU was influenced by computer self-efficacy, external control perceptions, anxiety, and enjoyment [55]. Chi and Ku [61] showed that voluntariness affected determinants of actual use, with EE increasing in importance as voluntariness decreased. Despite their importance, PU and PEOU may be undervalued in their significance for predicting BIs, especially when contextualized within newer constructs.

### UTAUT Framework and Critique

UTAUT consistently demonstrated stronger predictive performance than TAM. Shiferaw and Mehari [53] reported that UTAUT explains up to 70 % of the variance in the intention-to-use technology. However, Bagozzi [91] critiqued the model in that it coheres the “many splinters of knowledge” to explain decision-making and its subsequent extensions, stating “UTAUT is a well-meaning and thoughtful presentation,” but “it presents a model with 41 independent variables for predicting intentions and at least 8 independent variables for predicting behavior.” Furthermore, Li et al [92] argued that UTAUT’s reliance on moderators complicates model interpretation and may artificially inflate *R*² values. They also criticized construct labeling practices that combine disparate items into a single psychometric measure.

Nevertheless, UTAUT adds self-efficacy, social influence, and FCs, replacing TAM’s PU and PEOU with PE and EE. For example, one research paper found that self-efficacy was a significant predictor variable in comparing urban, rural, and remote settings in the Philippines [49], indicating that one’s ability to succeed or accomplish a task significantly and positively increased the intention to use an EHR in that setting. FC is a direct and commonly significant determinant of UB in the UTAUT framework with many subfactors [91-95].

### EHR Usability and Related Frameworks

Several tools and frameworks have been developed to assess EHR usability. A few papers use the SUS and NASA-TLX [69,79]. However, this review identified no studies that incorporated ISO-based frameworks [96] as evaluation or predictive factors for EHR usability or adoption.

There was mention of the Health Information Technology Usability Evaluation Scale. This scale is specifically designed for evaluating the usability of health IT applications, including EHRs. It includes subscales for measuring PU, PEOU, and user control, among others [37]. Additionally, Carayon et al [97] proposed the SEIPS model, which provides a framework for understanding the relationships between technology, work processes, and patient outcomes in health care settings. This review identified a study by Alsyouf and Ishak [79] that used SEIPS to predict and evaluate the usability of EHR systems by identifying potential mismatches between the technology and the users’ workflows.

The regression model by Butler and Johnson [60] did not predict EHR usability but rather productivity, which may be related to usability. Butler and Johnson [60] used an estimation framework for a productivity index based on physician characteristics, EMR functions, and vendors. Another study by Sulley and Ndanga [71] predicted US among physicians and different EHR system types or vendors and found that the EHR system type was significant. In addition, this study suggests that some EHR vendors can have higher satisfaction levels, which could be linked to the Butler and Johnson estimation of productivity using EHR. Moreover, Yin et al [73] used a logistic regression model and found that participants who are more likely to consider EHR portals as valuable tools were those who found EHR portals useful for information.

Therefore, a regression model of productivity with usability factors and EHR components across vendors could be an alternative for predicting usability instead of adding external usability variables to more common EHR adoption frameworks, such as constructs of TAM, UTAUT, and D&M ISS models. Furthermore, cognitive aspects, such as the theory of reasoned action, theory of planned behavior, and a user’s motivation to use IT, are influenced by the system features and capabilities in TAM, UTAUT, or D&M ISS model constructs. Further investigation is needed to predict EHR usability based on productive HCIs, the incorporation of aspects of EHR adoption frameworks, and other related models, such as Health Information Technology Usability Evaluation Scale, human factors engineering, SEIPS, and many more.

### Future Work

Emerging technologies, such as AI, machine learning, and advanced data mining, offer opportunities to extend these models and utilize large datasets. There is almost no research using AI with TAM, UTAUT, and D&M ISS.

The EHR adoption models initially emerged several decades ago. AI algorithms could add feedback loops or feedforward recursive networks to adjust to different data over time, for example, sequences of tasks, usability, and actual use of EHRs under different scenarios. Aspects of EHR usability could be added to the TAM and UTAUT models with training and test datasets via machine learning potentially integrated within SEM and CFA frameworks. Machine-learning techniques have been applied to the usability of health information systems [98].

Integrating audit log data, clickstream analyses, and HCI measures can enable predictive modeling of usability performance, informing adaptive design and proactive system improvements. There is evidence of increasing use of EHR audit log files as evidence for the macrostructure of EHR work completed [99]. Furthermore, there is a viewpoint that there are emerging domains for measuring health care delivery with EHR metadata: (1) team structure and dynamics, (2) workflows, and (3) cognitive environment [100]. These domains contain modified index or scoring, continuity index, comprehensiveness index, conformity score, undivided attention, and task switching [100].

Furthermore, Rule et al [34] stated that metadata from EHR from audit logs can be used to predict physician burnout, which could be considered a usability metric. Moreover, even if there are more recent studies that utilize metadata from EHR system logs, a major limitation is the absence of explicit task-level variables via large datasets to predict usability over time. There is, nevertheless, capacity to include such task measures in SEM-based models as determinants of PE, self-efficacy, EE, and traditional TAM constructs such as PU and PEOU.

Therefore, researchers should consider combining established adoption frameworks with continuous usability monitoring and predictive analytics. Predictive approaches—leveraging design attributes, certification data, usage patterns, and organizational indicators—could enable early identification of usability risks, when redesign and mitigation remain feasible. Furthermore, predicting EHR usability could be used for analyzing large datasets now being collected on EHR usability, anticipating usability problems with specific systems and supporting selection and procurement decisions for acquiring more usable EHRs. The work in this paper could form the groundwork for establishing future efforts in analyzing and predicting usability. Future work could develop feedback loops that link UB, system tasks, and usability outcomes, providing actionable insights for EHR vendors and health care organizations.

### Conclusion

No study in this review developed a predictive model for EHR usability postimplementation only, and very few studies used AI to predict adoption. To advance both EHR adoption and EHR usability sciences, researchers could bridge gaps between static adoption models and dynamic human factors engineering by integrating AI, audit log analysis, and real-time usability testing.

## Supplementary material

10.2196/86076 Multimedia Appendix 1 Table S1. Critique of 47 journal articles (2009-2025) reviewed and based on analytical techniques, metrics, and predictive findings and factors critique.

10.2196/86076 Multimedia Appendix 2 Frequency of usability metric.

10.2196/86076 Multimedia Appendix 3 Frequency of studies per year from the literature review.

10.2196/86076 Multimedia Appendix 4 Frequency of studies per country from the literature review.

10.2196/86076 Multimedia Appendix 5 Most frequent keywords in the research articles from the literature review.

10.2196/86076 Multimedia Appendix 6 Predictive analytic techniques of the 47 studies of the literature categorized into 12 data mining categories: classification and machine learning, correlation and association, dimensionality reduction and factor methods, measurement and validation methods, model evaluation and fit testing, qualitative and mixed methods approaches, regression methods, rank-based test for group comparison, resampling and robustness methods, structural equation modeling and latent constructs, and time series analysis. Many studies had more than 1 predictive analytic technique related to data mining.

10.2196/86076 Multimedia Appendix 7 Number of different techniques of predictive analytics across selected literature. The initially derived list of techniques and respective categories is from Multimedia Appendix 2. SEM: structural equation modeling.

10.2196/86076 Checklist 1 PRISMA-ScR checklist.

## Abbreviations

AI: artificial intelligence
BI: behavioral intention
CFA: confirmatory factor analysis
EE: effort expectancy
EHR: electronic health record
EMR: electronic medical record
FC: facilitating condition
HCI: human-computer interaction
IQ: information quality
ISO: International Organization for Standardization
ISS: DeLone and McLean information system success
JBI: Joanna Briggs Institute
NASA-TLX: National Aeronautics and Space Administration-Task Load Index
NuHISS: National Usability-Focused Health Information System Scale
PE: performance expectancy
PEOU: perceived ease of use
PRISMA-ScR: Preferred Reporting Items for Systematic Reviews and Meta-Analyses extension for Scoping Reviews
PU: perceived usefulness
SEIPS: Systems Engineering Initiative for Patient Safety
SEM: structural equation modeling
SQ: system quality
SRQ: service quality
SUS: system usability scale
TAM: technology acceptance model
TMTA: Tripolar Model of Technology Acceptance
TURF: task, user, representation, and function
UB: user behavior
US: user satisfaction
UTAUT: unified theory of acceptance and use of technology
