# Supplementary material for: Predicting Electronic Health Record Usability: Scoping Review of Adoption Models, Metrics, and Future Directions
Source: JMIR Hum Factors. 2026 Apr 8;13:e86076. doi: 10.2196/86076 (PMC13060746; doi:10.2196/86076)
Supplement: Multimedia Appendix 1 [file humanfactors-v13-e86076-s001.docx]

Multimedia Appendix 3

Table 2. Critique of 47 journal articles (2009-2025) reviewed and based on analytical techniques, metrics, and predictive findings and factors critique.

| **Authors** | **Study Design and Model Frameworks** | **Modelling Techniques** | **Usability Metrics** **and Psychometric Determinants** | **Significant Factor and Findings** | **Critique of Factors and Determinants** |
| --- | --- | --- | --- | --- | --- |
| [45] Abdekhoda, M.; Ahmadi, M.; Dehnad, A.; Hosseini, A.F. (2014). | Descriptive–analytical study of IT acceptance using Technology Acceptance Model (TAM) of health information management in Tehran, Iran. | Regression analysis examining perceived ease of use (PEOU), perceived usefulness (PU), and Attitude. | Attitude toward using IT; PEOU; PU. | PU and PEOU were positively associated with favorable attitudes toward HIM systems. | No measurement of task performance (time on task, errors, task success rate) to confirm whether ease of use is reflected in real workflows. |
| [46] Addo, K.; Agyepong, P.K. (2024). | Mixed-methods using TAM evaluated the implementation EHR and District Health Information Management System (DHIMS-2) in Ghana. | Pearson’s correlation between PEOU, PU, Attitude, and Actual Usage. Linear & multiple regression analysis tested TAM predictions on system acceptance. | Attitude toward using IT; Frequency of Use; Intention to use; PEOU; Perceived benefits (e.g. reduced workload, improved patient wait times, claims processing); PU; System Use; User Satisfaction (US). | Integrates TAM with affective variables (positive and negative emotions), providing richer insights into acceptance. PU and attitude towards intention to use were significant factors (61% variance explained). | PEOU did not predict attitude, which is inconsistent with TAM theory. |
| [47] Akwaowo, C.D.; Sabi, H.M.; Ekpenyong, N.; Isiguzo, C.M.; Andem, N.F.; Maduka, O.; Dan, E.; Umoh, E.; Ekpin, V.; Uzoka, F.-M. (2021). | Clinicians and senior management in healthcare facilities of the adoption of EMR using extended TAM in developing countries, particularly in the Niger Delta region of Nigeria. | Exploratory factor analysis (EFA) extracted and validated constructs influencing EMR adoption. | Awareness and Training; PEOU; PU. | Usefulness, critical success factors, awareness, and relative advantages positively influence EMR adoption. | PEOU was not significant compared to usefulness and risk concerns, but lack of significance weakens the model’s strength as a usability predictor for Extended TAM. |
| [48] Almarzouqi, A.; Aburayya, A.; Salloum, S.A. (2022). | Integrated approach using TAM and Unified Theory of Acceptance and Use of Technology (UTAUT) questionnaire among providers in Dubai, United Arab Emirates (UEA). | Partial Least Square – Structural Equation Modelling (PLS-SEM) analyzed the relationships between different constructs. Artificial Neural Networks (ANN) confirmed PLS-SEM analysis results. Variance Inflation Factor (VIF) examined any difficulty with linearity and MSE for model accuracy. | Effort Expectancy (EE); PEOU; PU; Social Influence (SI). | SI, SE, PE, and PU significantly impacted EMR adoption. Convergent validity determined by average variance extracted (AVE). IPMA findings of management were divided into performance and significance. | PLS-SEM and ANN combined formed a non-linear predictive model for understanding EMR adoption. EMR is a black box in this study — does not reveal which screens, tasks, or modules (CPOE, charting) were usability bottlenecks. |
| [49] Alsohime, F.; Temsah, M.-H.; Al-Eyadhy, A.; Bashiri, F.A.; Househ, M.; Jamal, A.; Hasan, G.; Alhaboob, A.A.; Alabdulhafid, M.; Amer, Y.S. (2019). | Explored perceptions of physicians working in pediatrics at King Saud University Medical City, Riyadh, Saudi Arabia. | Bivariate and multivariate regression identified factors with physicians’ satisfaction with principal component analysis (PCA) and confirmatory factor analysis (CFA). | Availability of Computers; Individual and Team Performance; PEOU; PU; Satisfaction with EHR; Time Efficiency. | Ability to reduce errors and enhanced individual performance were top indicators. | Usability inferred through perception, no behavioral data. PEOU dimension was buried under satisfaction. |
| [50] Alsyouf, A.; Ishak, A.K. (2018). | Mixed-method and heuristic evaluation of EHRs continuance intention to use from perspectives of UTAUT in Jordanian hospitals. | System Usability Scale (SUS); NASA-Task Load Index (TLX); association rule mining analyzed navigation patterns and usage; decision-tree; heuristic evaluation. | Heuristic evaluation scores based on usability principles; NASA-TLX; SUS. | Higher workload, lower usability post-implementation; Significant differences in workload and usability by clinician role (higher for providers). Cognitive workload predicted by the TAM and SEIPS model. | TAM and SEIPS predicted workload and usability. Model lumps the EHR into one block — so it cannot pinpoint which modules (e.g., order entry, charting) have good or poor usability. No task/module-specific insights. |
| [51] Beglaryan, M. (2017). | Multi-stage cluster sampling of physicians using Tripolar Model of Technology Acceptance (TMTA**)** in hospitals in Yerevan, Armenia. | Exploratory SEM with a robust weighted-least-squares estimator for categorical indicators. | Patient Influence; Personal Innovativeness; Projected Collective Usefulness; Resistance to Change. | TMTA explained 85% of variance in the behavioral intention to use technology. | TMTA predicts general behavioral intention but does not map how specific EHR features (e.g., CPOE, documentation screens) perform in practice. |
| [52] Bossen, C.; Jensen, L.G.; Udsen, F.W. (2013). | Formative evaluation of EHR using DeLone and McLean (D&M) model for Information System Success (ISS) at a regional hospital in Denmark. | ANOVA tests, qualitative analysis coding, process control. | Information Quality (IQ); System Quality (SQ); Service Quality (SRQ). | Positive EHR reliability and response time; cumbersome administration module. | D&M ISS applied; mandatory use limits usability interpretation. |
| [53] Butler, R.J.; Johnson, W.G. (2016). | Physician perceptions of EMR systems in Arizona State University, USA. | Multivariate regression models on the perceived usability and productivity of the EMRs. | PEOU; Performance vs. Vendor Promises; Productivity; Reliability. | Physician and practice characteristics influenced perceived productivity and usability. | Constructs conflate usability with productivity: EHR can be easy to use but still inefficient if workflows are poorly matched. Task-level usability was not measured. |
| [54] Campione, J.; Liu, H. (2024). | Survey of 8,880 hospital staff from 44 hospitals in USA. | ANOVA with Tukey-Kramer post-hoc tests for mean/frequency differences across groups. Mixed-effects regression models; Logistic regression models. General estimating equation (GEE) used for clustering by hospital units when modeling safety perceptions. | Information Accuracy; Training/Support; US; Workflow. | 20% dissatisfied with EHR. Top-related issues were hard-to-find important information (28%) and information incomplete (25%). | Large sample but limited by self-report, bias in negative perceptions (confounding) of EHR generally may influence safety responses, and lack of vendor/system data. |
| [55] Chiu, T.M.L.; Ku, B.P.S. (2019). | Effects of voluntariness on the actual use of an EHR system using UTAUT by allied health professionals in Hong Kong, China. | Multiple logistic regression conducted to identify factors associated with the actual use. | Attitude towards technology (Confidence, Approval, Interest); EE; Facilitating Condition (FC); PE; SI. | PE, FC, and SI predicted use in non-mandatory or high voluntariness environment. | EE was used as an independent variable, not a direct usability index. Usability confounded with task allocation. |
| [15] Dimitrovski, T.; Bath, P.A.; Ketikidis, P.; Lazuras, L. (2021). | General practitioners surveyed in North Macedonia (Thessaloniki, Greece) using a modified UTAUT model. | Hierarchical linear regression with intentions to use EHR system. Multiple mediation modeling analysis determined the effects of EE and SI. Regression analysis evaluated the effects of age and experience. | Descriptive Norm (DN); EE; FC; Job Relevance (JR); SI. | EE, SI, FC, DN, and JR significant factors. Performance expectancy was not significant. | Future readiness for a proposed system was tested — not usability experience of EHR. |
| [56] Dubale, A.T.; Mengestie, N.D.; Tilahun, B.; Walle, A.D. (2023). | User satisfaction with electronic medical records (EMR) systems among health professionals in Addis Ababa, Ethiopia. | Bivariable and multivariable logistic regression analyses identified user satisfaction with the EMR system. | Computer Access; Computer Literacy; eHealth Literacy; EMR Training; IQ; SQ; SRQ. | EMR training, computer literacy, SQ, IQ, SRQ, and access to computers as significant determinants. | Self-reported only. No measurement of task performance, error rate, time-to-complete tasks, or ease of learning — that are all standard usability criteria (ISO 9241-11). |
| [57] Ebnehoseini, Z.; Tara, M.; Tabesh, H.; Dindar, F.H.; Hasibian, S. (2020). | Using TAM3 to investigate factors affecting the acceptance of hospital EHR adoption by users in Iran. | Multiple linear regression. | Behavioural Intention (BI); PEOU; PU; Use Behavior (UB). | BI, PEOU, BI, PU, and UB influenced EHR adoption. | PEOU and PU were not significant on actual use behavior. Only behavioral intention predicted use. TAM3 was better at predicting intentions than usability and performance. |
| [16] Gagnon, M.P.; Ghandour, E.K.; Talla, P.K.; Simonyan, D.; Godin, G.; Labrecque, M.; Ouimet, M.; Rousseau, M. (2014). | Physician acceptance of EHR using four theoretical models including TAM in Quebec University Hospital, Canada. | Path analysis and multiple linear regression analysis tested the four theoretical models and identify the main determinants. | Demonstrability of results; PEOU; Professional Norm; Resistance to Change; PU; Self Efficacy (SE); SN. | Integrated model performed best in explaining physicians' intention to use EHR: 55% of the variance. | Extended TAM, psychosocial models, and additional constructs to predict physicians' acceptance of EHRs. The model does not identify which modules (e.g., charting, labs, prescribing) drive poor PEOU or PU. |
| [58] Gilani, M.S.; Iranmanesh, M.; Nikbin, D.; Zailani, S. (2017). | Acceptance of EMRs among healthcare professionals in multiple hospitals in Iran. | PLS-SEM tested the research model and analyzed relationships among constructs in TAM, ECM, and COGM. | Attitude; PEOU; PU; Satisfaction. | Attitude, PU and US were significant. | Technology Continuance Theory (TCT) was used to predict whether users keep using the EMR — not whether the EMR was usable in ISO 9241-11 terms (effectiveness, efficiency, and satisfaction). |
| [59] Hadji, B.; Martin, G.; Dupuis, I.; Campoy, E.; Degoulet, P. (2016). | Over 14 years analyzed a Clinical Information System (CIS) by healthcare professionals including TAM at the Georges Pompidou European University Hospital in Paris, France. | Multiple regression analysis explored the relationships between different acceptance dimensions. SEM applied to test CIS quality, confirmation of expectations, global satisfaction, and PU. GEE used for system acceptance. | CIS Quality; CIS Use; Confirmation of Expectations; FC; Global Satisfaction; PU. | User satisfaction initially linked to CIS use, but later factors like SQ and confirmation of expectations. Reduction in the relationship between CIS use and satisfaction over time. | No time-on-task, task success rate, or error tracking — usability prediction remains perceptual, not performance-based. |
| [60] Hyppönen, H.; Kaipio, J.; Heponiemi, T.; Lääveri, T.; Aalto, A.-M.; Vainiomäki, J.; Elovainio, M. (2019). | Finnish physicians validated the National Usability-Focused HIS Scale (NuHISS) in Helsinki, Finland. | EFA of the factor structure of NuHISS items. SEM was used for confirmatory factor analysis (CFA) to test validity and reliability. | Benefits; Cross-Organizational Collaboration; Feedback; Internal Collaboration; IQ; PEOU; Technical Quality. | NuHISS was a valid and reliable tool for monitoring long-term development. | Despite being detailed, NuHISS relies entirely on subjective self-reports. While it covers effectiveness, efficiency, satisfaction (ISO 9241-11 core usability aspects), it does not measure learnability or memorability directly. |
| [61] Iqbal, U.; Ho, C.H.; Li, Y.C.J; Nguyen, P.A.; Jian, W.S.; Wen, H.C. (2013). | Usage intention and adoption behavior of EHRs among primary care physicians in Taiwan using TAM. | Chi-squared analysis and independent *t*-tests compared adopters versus non-adopters. Multivariate logistic regression identified factors with EHR adoption. | Intention to Use (IU); PEOU; PU; SE; Security and Privacy Concerns; SN. | Physicians' intention to use EHRs, PEOU and PU were crucial factors. Privacy concerns and SN negatively impacted the adoption. | The outcome was adopter versus non-adopter — not a measure of how usable the EHR is in workflows once adopted. |
| [62] Kaihlanen, A.-M.; Gluschkoff, K.; Hyppönen, H.; Kaipio, J.; Puttonen, S.; Vehko, T.; Saranto, K.; Karhe, L.; Heponiemi, T. (2020). | EHR usability and user age with stress related to information systems and cognitive failures among Finnish registered nurses using NuHISS. | Multiple linear regression analysis explored the relationship between EHR usability, age, stress, and cognitive failures. GEE (logistic + linear) to model stress with EHR usability. | Cognitive Failures; Information System-Related Stress; PEOU; System Usability. | Both younger and older nurses experienced stress, but younger nurses had more cognitive failures when they found the EHR difficult to use. | Model tests how perceived usability predicts stress related to information systems and cognitive failures. This directly connects poor usability to practical, measurable consequences for workflow and safety. |
| [63] Ketikidis, P.; Dimitrovski, T.; Lazuras, L.; Bath, P.A. (2012). | TAM of Health Information Technology (HIT) in three clinics in Skopje, North Macedonia, and Thessaloniki, Greece. | Multiple linear regression analysis was used to determine the predictors of HIT acceptance. | Computer Anxiety; Descriptive Norms (DN); Job Relevance (JR); PEOU; PU; SN. | TAM variables were critical, but PU did not significantly predict HIT usage intentions. | Respondents were asked to imagine using HIT, rather than assessing an actual EHR they work with. This weakens ecological validity: people may misestimate ease of use when they have no direct system experience. |
| [64] Kutney-Lee, A.; Sloane, D.M.; Bowles, K.H.; Burns, L.R.; Aiken, L.H. (2019). | EHR adoption levels, work environment, and outcomes related to EHR usability and quality of care in Philadelphia, Pennsylvania, USA. | Logistic regression models of comprehensive EHR adoption and outcomes related to EHR usability and nurse-reported quality of care and patient safety. GEE analyzed nurses nested within hospitals. | Accuracy of Information; Ease of Access to Patient Data; Impact on Work Efficiency; Information Sharing Capability; Nurse Satisfaction with EHR; PEOU. | Comprehensive EHR adoption is associated with lower odds of nurses reporting poor usability outcomes. Nurses in better environments were less likely to report negatively. | Adoption level is structural, not functional usability. Comprehensive EHR means the system has more modules (e.g., CPOE, barcoding, decision support) but the model does not break down which features improve usability vs. increase burden. |
| [65] Kutney-Lee, A.; Brooks Carthon, M.; Sloane, D.M.; Bowles, K.H.; McHugh, M.D.; Aiken, L.H. (2021). | EHR usability and nurse Job outcomes and surgical patient outcomes in Philadelphia, Pennsylvania USA. | Logistic regression models of EHR usability and outcomes (nurse and patient outcomes) before and after an EHR adoption level (comprehensive vs. basic or less). GEE for multi-level survey of nurses nested in hospitals. | Burnout; Efficiency; EHR usability; Nurse-reported EHR usability; PEOU; System Interference. | Nurses working in hospitals with poorer EHR usability had increased burnout. Surgical patients in hospitals with poorer EHR usability had higher mortality and 30-day readmission. | Analysis was cross-sectional so causality cannot be proven. For example, high burnout could make nurses rate usability lower, not vice versa. There is no task-based observation: no data on actual time-on-task, navigation clicks, or error rates. |
| [18] Lee, S. (2022). | Modified D&M ISS model of an EMR on nurses’ perceptions in hospitals in South Korea. | Hierarchical multiple regression analysis determined the effect of system quality, information quality, and service quality on PU and PEOU. | IQ; SRQ; SQ. | Study demonstrated SQ, IQ, and SRQ significant factors influencing nurses' PU and PEOU of EMR systems. | The model clarifies which quality factors affect PEOU, but it does not show which concrete design changes would improve usability scores. |
| [66] Lopez, K.D.; Chin, C.-L.; Azevedo, L.R.F.; Kaushik, V.; Roy, B.; Schuh, W.; Banks, K.; Sousa, V.; Morrow, D. (2021). | Mixed-method approach, to identify usability issues in the EHR system in Iowa City, USA. | Self-administered surveys and workload with SUS and NASA-TLX. Heuristic evaluation for evaluating usability. GEE to assess changes over time in workload and usability. | NASA Task Load Index; System Usability Scale (SUS). | Workload increased immediately after EHR adoption and usability decreased. | Clinician factors with safety based on the TAM and the Systems Engineering Initiative for Patient Safety (SEIPS) model​. The model tests whether lower usability predicts higher workload (NASA-TLX) confirming usability matters for real cognitive burden. |
| [67] Mesa, R.Y.H.; Galingana, C.L.T.; Tan-Lim, C.S.C.; Javelosa, M.A.U.; Panganiban, J.M.S.; Fabian, N.M.C.; Calderon, Y.; Rey, M.P.; Bernal-Sundiang, N.; Sanchez, J.T.; Dans, L.F.; Casile, R.U.; Dans, A.L. (2024). | User acceptance of locally developed Open Medical Records System evaluated in rural and urban areas using UTAUT in the Philippines. | Ridge regression predictors of behavioural intention (BI) to use EHR. | Anxiety (AN); Attitude; BI; EE; FC; PE; SE; SI. | Self-efficacy was the only domain significantly associated with BI. | UTAUT framework allowed comprehensive examination of BI predictors. Inclusion of three diverse settings (urban, rural, remote) enhances contextual understanding. Self-reported data may be subject to social desirability bias. No baseline pre-EHR data to measure change in efficiency or performance. |
| [68] Messeri, P.; Khan, S.; Millery, M.; Campbell, A.; Merrill, J.; Shih, S.; Kukafka, R. (2013). | Using D&M ISS model to evaluate EHR use of primary care providers in New York, USA. | Principal Factor Analysis; PCA; and Multiple Regression. | Information System Use (ISU); PEOU; SQ; US. | SQ, IS, PEOU, and US were highly intercorrelated. | Model does not include an explicit usability construct. The model relies solely on PEOU, not on any observational or objective usability data. |
| [69] Mijin, N.; Jang, H.; Choi, B.; Khongorzul, G. (2019). | Attitudes of medical professionals toward the use of EMR systems within the context of the TAM and Innovation Diffusion Theory (IDT) in South Korea. | CFA evaluated the measurement model. SEM tested the hypothesized relationships between the variables and assessed the paths between TAM constructs. | Accuracy; Compatibility; PEOU; PU; Reliability; Security; Self-Image. | Compatibility, security, and accuracy positively impacted PU, while reliability did not. Self-image moderates the relationships between PEOU and Attitude toward EMR use, as well as PU and attitude toward EMR use. | Their technical attributes (i.e. compatibility, security, accuracy, reliability) were user-reported, not measured. |
| [70] Morton, M.E; Wiedenbeck, S. (2010). | Physician attitudes toward adopting an EHR system in ambulatory care at University of Mississippi Medical Center in Jackson, Mississippi, USA. | Multiple regression analyses assessed relationships among physician characteristics, computer experience, and EHR acceptance factors. SEM used to predict physician attitudes toward EHR adoption. | Doctor-Patient Relationship; PEOU; Physician Autonomy; Physician Involvement; PU; Management Support; Training Adequacy. | Sociotechnical factors, rather than individual physician characteristics, played a significant role in shaping attitudes toward adoption. | While PEOU was included, it was treated as an independent predictor of Attitude — not as a stand-alone usability outcome. There are no task-based measures: no data on task completion times, error rates, or workflow disruptions. |
| [71] Mwogosi, A.; Kibusi, S. (2024). | TAM3 used to assess and predict EHR usability among healthcare workers from 17 primary healthcare facilities in Tanzania. | Multiple linear regression assessed the impact of decision support system (DSS) features and usage frequency on clinical decision-making. Thematic analysis and word cloud visualizations identified recurring terms. | BI; Decision Support Effectiveness; FC; Frequency of Use; PEOU; PU; Subjective Norms (SN); US; Usability Ratings. | User satisfaction had negative significant impacts. No significant relationship with clinical decision making and EHR features or usage frequency. EHR is seen as moderately effective for documentation and workflow efficiency. | Negative relationship between DSS features/frequency of use and satisfaction suggests TAM3 may need augmentation with usability burden or workflow fit constructs. Model did not capture all the factors influencing clinical decision-making — other organizational and environmental variables could be more critical. |
| [72] Napitupulu, T.A.; Julham Patria, S.H. (2013). | Modified UTAUT identified factors influencing user satisfaction with EMR systems in hospitals in Indonesia. | SEM done in two steps: (1) CFA validated the operational variables, and (2) multiple regression analysis tested impact of the independent variables on user satisfaction. | EE; FC; IQ; Organizational Culture (OC); PE. | All five factors— PE, EE, IQ, FC, and OC—had a positive influence on the level of user satisfaction with the EMR system. | The study does not measure real-world task efficiency, error rates, or learnability. Snapshot only does not test how usability perceptions change as the system matures, or upgrades are made. |
| [73] Rhayha, R.; Ismaili, A.A. (2024). | Modified D&M ISS model in Moroccan university hospitals. | Exploratory factor analysis (EFA), confirmatory factor analysis (CFA). Large, combined sample size (N=532) and separation of EFA and CFA samples to reduce bias. | Clinical Performance (CP); IQ; IT Service Quality (ISQ); Organization (ORG); Environment (ENV); SQ; US. | 28-item validated instrument with seven constructs. EFA explained 68% of variance. US had strongest correlation. | Single-site study limits generalizability. Focused only on current EHR functions; did not address broader EHR-related tasks (research, audit, financial benefits, workload, resistance to change). |
| [74] Sachdeva, S.; Madaan, A.; Bhalla, S. (2012). | Enhancing the usability of EHR systems by discovering patterns in user behavior and needs through data mining techniques in Japan. | Association analysis found patterns in user navigation and identify common tasks and needs. Classification and prediction categorized users based on demographics and predict their future actions. | Effectiveness; Efficiency; Error Recovery; Learnability; PEOU. | High accuracy in classifying user categories and predicting user actions, with reduced errors, and improved efficiency in user interactions. | Model assumes high-quality usage logs exist and are rich enough for mining — but many EHRs still lack detailed clickstream or event logs. No actual task completion time, error rates, or efficiency tests on real EHR tasks. |
| [75] Sharifian, R.; Askarian, F.; Nematolahi, M.; Farhadi, P. (2014). | Factors influencing the acceptance in UTAUT model among nurses in hospitals affiliated with Shiraz University of Medical Sciences in Shiraz University of Medical Sciences, Shiraz, Iran. | SEM was employed using LISREL software to estimate path coefficients and test hypotheses. | BI; EE; FC; PE; SI. | PE was the most significant predictor of behavioral intention, followed by FC, SI, and EE. | UTAUT model predicted nurses' acceptance and use of HIS, but EE was used as a predictor of BI, not as a stand-alone usability outcome. The study relies 100% on subjective self-reported PEOU — no measurement of real task performance, error rates, or time on task. |
| [76] Shiferaw, K.B.; Mehari, E.A. (2019). | Acceptance and use of the EMR system among doctors and nurses using UTAUT in hospitals in northern Ethiopia. | SEM explained the extent of the relationship among variables. CFA validated the measurement model, ensuring reliability and validity. | Attitude; EE; FC; PE; SE; SI. | EE, intention to use, FC, and SE significantly influenced the actual use of the EMR. | Attitude, EE, SE, and other constructs are self-reported. No task-based measurement (e.g., time-on-task, clicks, workflow bottlenecks) to test actual usability. |
| [77] Shudayfat, T.; Akyürek, Ç.E.; Al-Shdayfat, NM., Alsaqqa, H.H. (2024). | TAM extended with variables from usability theory, including user satisfaction, learnability, system performance, and perceived usefulness in Jordanian hospitals. | Correlation analysis to identify relationships between TAM constructs (PU, PEOU, attitudes) and organizational factors. | PEOU; PU. | PEOU and PU predicted task perceptions. Organizational factors (competency, management support, training & education) were significantly positively correlated with PU, PEOU, and Attitude. | Context-limited predictive value for clinical impact, data only from northern Jordanian hospitals. Organizational factors were self-assessed, not objectively measured. |
| [78**]** Sicotte, C.; Paré, G.; Moreault, M.-P.; Lemay, A.; Valiquette, L.; Barkun, J. (2009). | Assessed user satisfaction via inpatient medical record system before its replacement at two Canadian teaching hospitals in Montreal using D&M ISS model. | Multivariate regression analyses. Factor analysis verified dimensions of outcomes related to quality of care and patient safety. Used GEE (logit) for repeated user satisfaction ratings pre/post replacement. | IQ; Perceived Outcomes; PU. | PU correlated with system quality attributes, while the perceived impact on quality of care and patient safety showed weaker correlations. | Usability perceptions did not strongly predict clinical impact. Limited predictive value for clinical impact. |
| **[**79**]** Sintonen, S.; Mäkelä, K.; Miettinen, R. (2015). | TAM and the Theory of Planned Behavior (TPB) used to examine the acceptance of an EHR system by medical personnel in regional hospital in southeastern Finland. | PLS-SEM analyzed relationships between variables of system complexity, perceived behavioral control, and usage intention. | Perceived Behavioral Control; PU; System Complexity; System Reliability; Usage Intention. | System complexity and reliability problems negatively influenced the PU of the EHR system, reducing the willingness of healthcare personnel to use it. | Perceived control (skills/confidence) did not affect intention or use — so the social/skill branch in the model did not contribute meaningfully to predicting usability-related outcomes. |
| **[**80**]** Sulley, S.; Ndanga, M. (2025). | 2018–2021 National Electronic Health Records (NEHR) Survey from the National Center for Health Statistics (NCHS) in New York City, USA. | Interaction analysis by age group and EHR system type. Pearson’s r examined relationships between satisfaction and EHR features. Multivariate regression. | EHR Satisfaction Score; Feature-based Metrics (CPOE usage score, e-Prescribing usage score, customization score (practice-specific adaptation); Interoperability Score (integration with other systems). | Significant positive predictors: Epic, Cerner (Oracle Health), and Athenahealth vendors. Significant negative predictors: Allscripts and e-MDs. Feature effect by age: <50 yrs: CPOE significant and ≥50 yrs: e-prescribing significant. | Recall/social desirability bias possible. Overrepresentation of older physicians by 66% of sample potential bias. Acquisition by Oracle Health happened post-study so future satisfaction may differ, as mentioned by the authors of this journal article. |
| **[**81**]** Tilahun, B.; Fritz, F. (2015). | Usage pattern and user satisfaction, with EMR system implemented using D&M ISS model in Addis Ababa, Ethiopia. | Binary logistic regression analysis identified determinants of US. | EMR Use Rate; US. | Poor service quality, dual documentation and partial departmental usage related to dissatisfaction. Computer access, IT qualification, SQ, IQ, and SRQ were significant to satisfaction. | Workflow confounds usability measures, which undermined real usage and perceived usefulness — but that was partly an organizational problem in this study, not just a technical usability failure. |
| **[**82**]** Tilahun, B.; Fritz, F. (2015). | A quantitative study among health professionals in five public hospitals in Ethiopia using D&M ISS model. | SEM clarified relationships between variables. Used GEE to account for clustering of users within hospitals when modeling satisfaction scores. | EMR Use; IQ; Perceived Net-Benefit; SQ; SRQ; US. | SQ, IQ, and SRQ significantly influenced both EMR use and user satisfaction. | The EMR use construct was about frequency of use — which is influenced by mandates, policy, and habits — not purely by usability. |
| **[**17**]** Tubaishat, A. (2017). | Satisfaction of EHR of nurses using D&M ISS model in 17 hospitals in Jordon. | Multiple regression analysis identified predictors of nurses' responses concerning EHR use, quality, and satisfaction. | Quality of EHRs; Use of EHRs; US. | Factors such as age, type of record, comfort level with computers, and hospital size were significant. | No task-based metrics: no real measurement of time on task, error rates, navigation flow, or log files to test if high quality perceptions match practical usability in daily work. |
| **[**83**]** Tubaishat, A. (2018). | Exploring nurses’ perceptions of EHRs of TAM predictors in multiple hospitals in Jordan. | Multiple regression analysis assessed predictors of nurses' perceptions regarding the PU and PEOU of EHRs. | PEOU; PU. | Nursing experience, and computer skills were significant predictors for PEOU. | TAM predicted nurses' acceptance of EHRs based on PU and PEOU. The model stops at predicting acceptance — not whether the EHR was efficient, error-resistant, or improves real workflow outcomes. |
| **[**84**]** Turan, A.H.; Koç, T. (2022). | Using a modified TAM to assess the adoption and acceptance of EHR among Turkish physicians. | SEM and CFA explored relationships between different constructs and validated the model. | Commitment; Motivation; PEOU; PU; Resistance. | Physicians’ commitment to using HIT positively influenced their intention to use it. | Motivation (H1) and resistance (H3) were not significant predictors of intention to use in this setting — only commitment mattered. This questions if the expanded model constructs add meaningful predictive value for usability. |
| **[**85**]** Vainiomäki, S.; Aalto, A.-M.; Lääveri, T.; Sinervo, T.; Elovainio, M.; Mäntyselkä, P.; Hyppönen, H. (2017). | Evaluated relationship between electronic patient record (EPR) usability and work-related well-being of physicians in Finland. | EFA used to group the usability items into seven dimensions to assess. Hierarchical multivariate regression analyses. GEE (logistic + linear) for physicians clustered by organization to relate usability and well-being. | Ease of Access to Test Results; External Cooperation; Feedback; Internal Cooperation; Perceived Benefits; Technical Problems; User Friendliness. | Easy readability of nursing records was linked to higher job control and lower time pressure. | Dependent variables were time pressure and job control — these were outcomes influenced by usability, but not direct usability measures. The prediction was limited to statistical associations with well-being, not predictive performance on usability tasks. |
| **[**86**]** Yin, R.; Neyens, D.M.; Law, K.E. (2020). | 500 U.S. respondents recruited via Amazon Mechanical Turk (MTurk) to identify how users perceive their EHR patient portal. | Logistic regression. Stepwise Akaike Information Criterion (AIC) to select the best-fit model. | Importance; Decision-making Support; Habitual Use; PEOU; PU; Time to become familiar; Trust. | PEOU, habitual use, and frequent internet use – negative association. Low baseline metrics – only ~7% trusted their EHR portal. | Did not differentiate between specific EHR portal systems (e.g., EPIC MyChart vs. others) — limits applicability to system-specific design changes. Not task-specific measures — did not distinguish between general and health-related online activities. |
| [12] Zhang, J.; Walji, M.F. (2011). | TURF (Task, User, Representation, and Function) structured approach to improving EHR usability in Houston, Texas, USA. | Qualitative analysis. Task time, error rates, and user satisfaction were metrics used to objectively measure usability. | Error Rates; Satisfying; Task Time; Usable; PU; US. | TURF framework identified usability issues in EHR systems by focusing on tasks, users, representations, and functions. | Workflow interruptions, team hand-offs, multitasking, and social context heavily shaped EHR usability — but these are flagged for future expansion, not fully operationalized in the current TURF version. |
