# Supplementary figures and images for: Predicting Electronic Health Record Usability: Scoping Review of Adoption Models, Metrics, and Future Directions
Source: JMIR Hum Factors. 2026 Apr 8;13:e86076. doi: 10.2196/86076 (PMC13060746; doi:10.2196/86076)

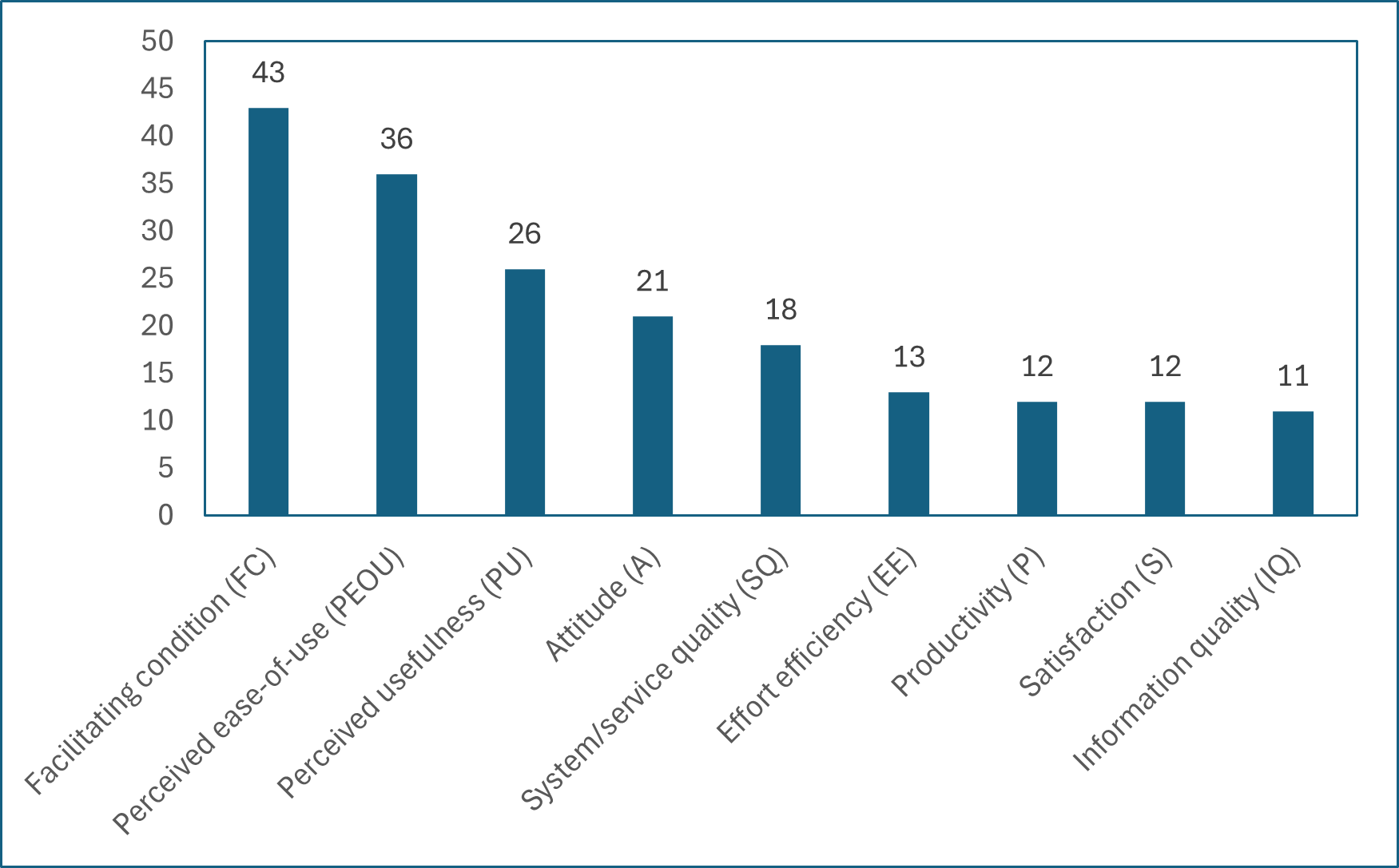

Supplement: Multimedia Appendix 2 [file humanfactors-v13-e86076-s002.png]

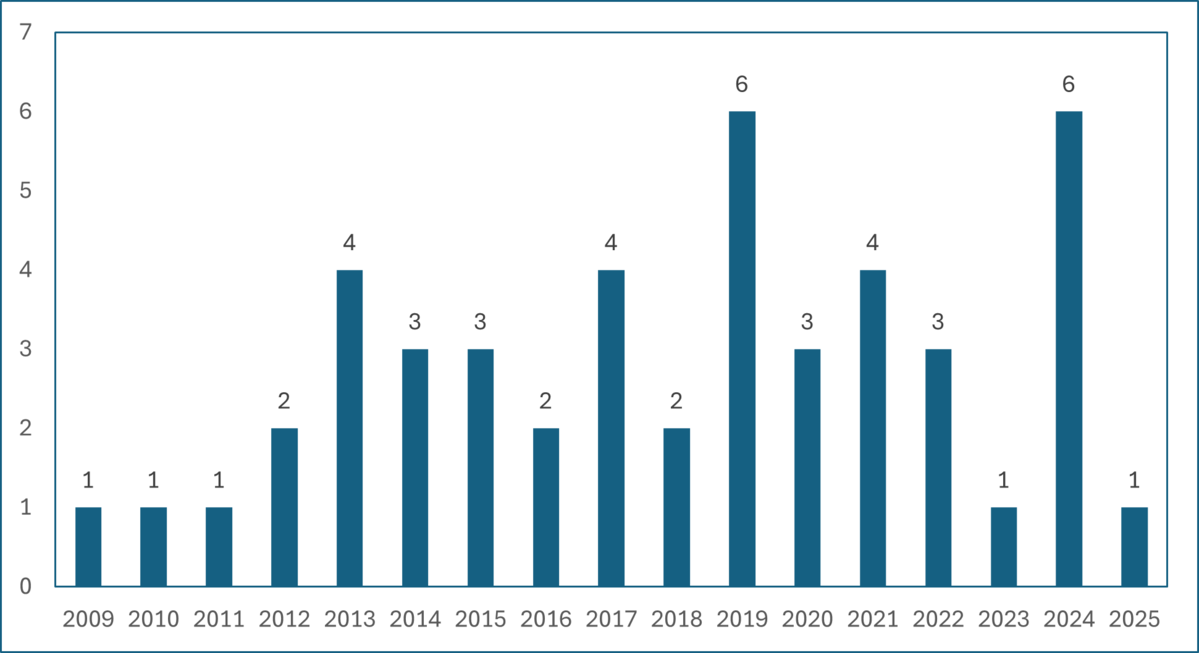

Supplement: Multimedia Appendix 3 [file humanfactors-v13-e86076-s003.png]

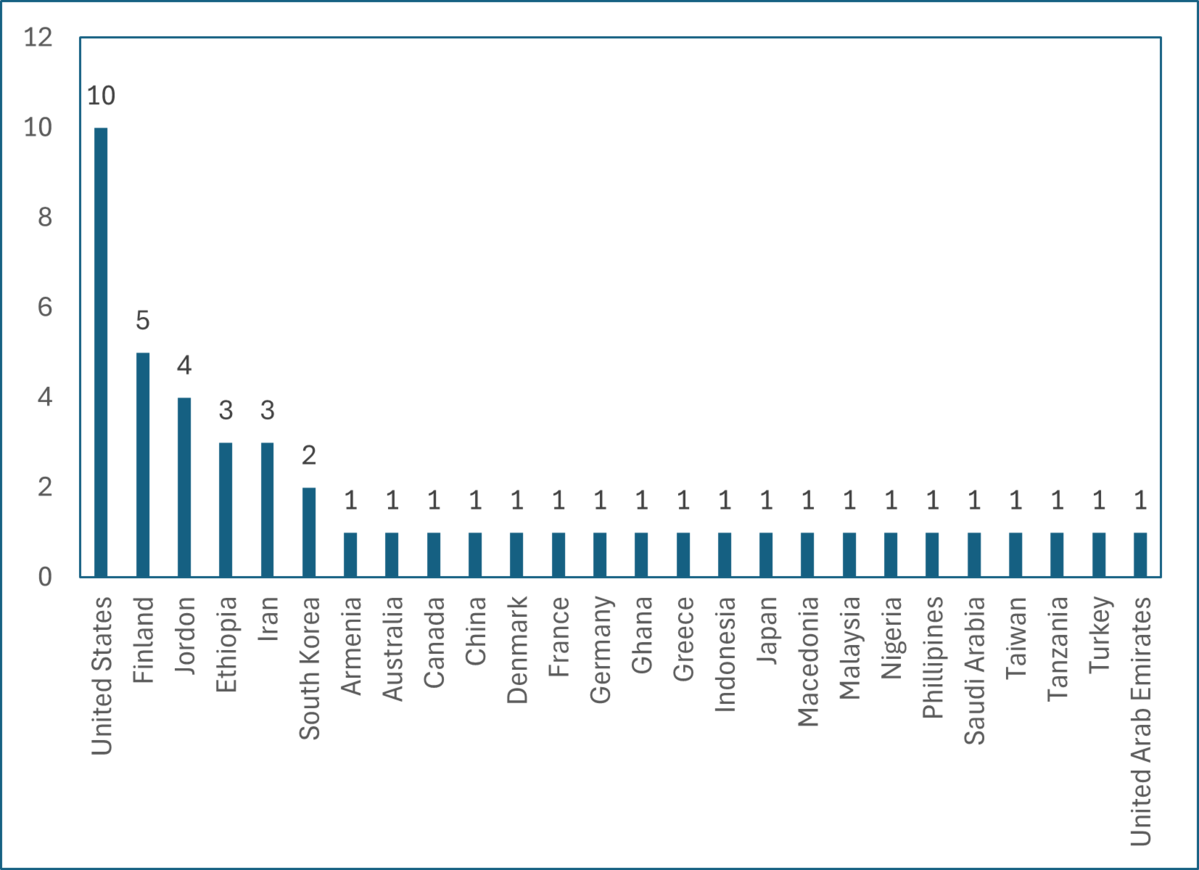

Supplement: Multimedia Appendix 4 [file humanfactors-v13-e86076-s004.png]

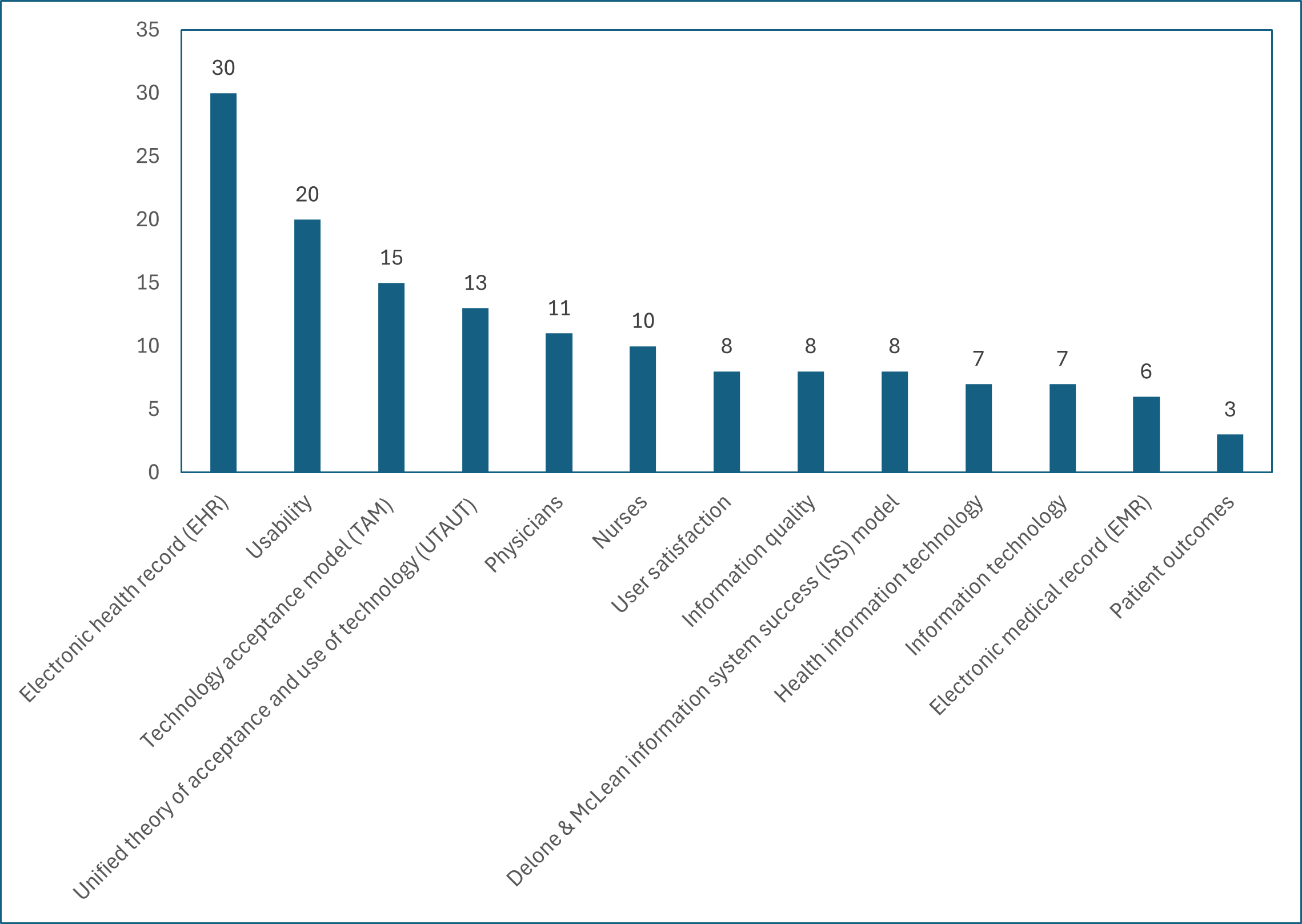

Supplement: Multimedia Appendix 5 [file humanfactors-v13-e86076-s005.png]

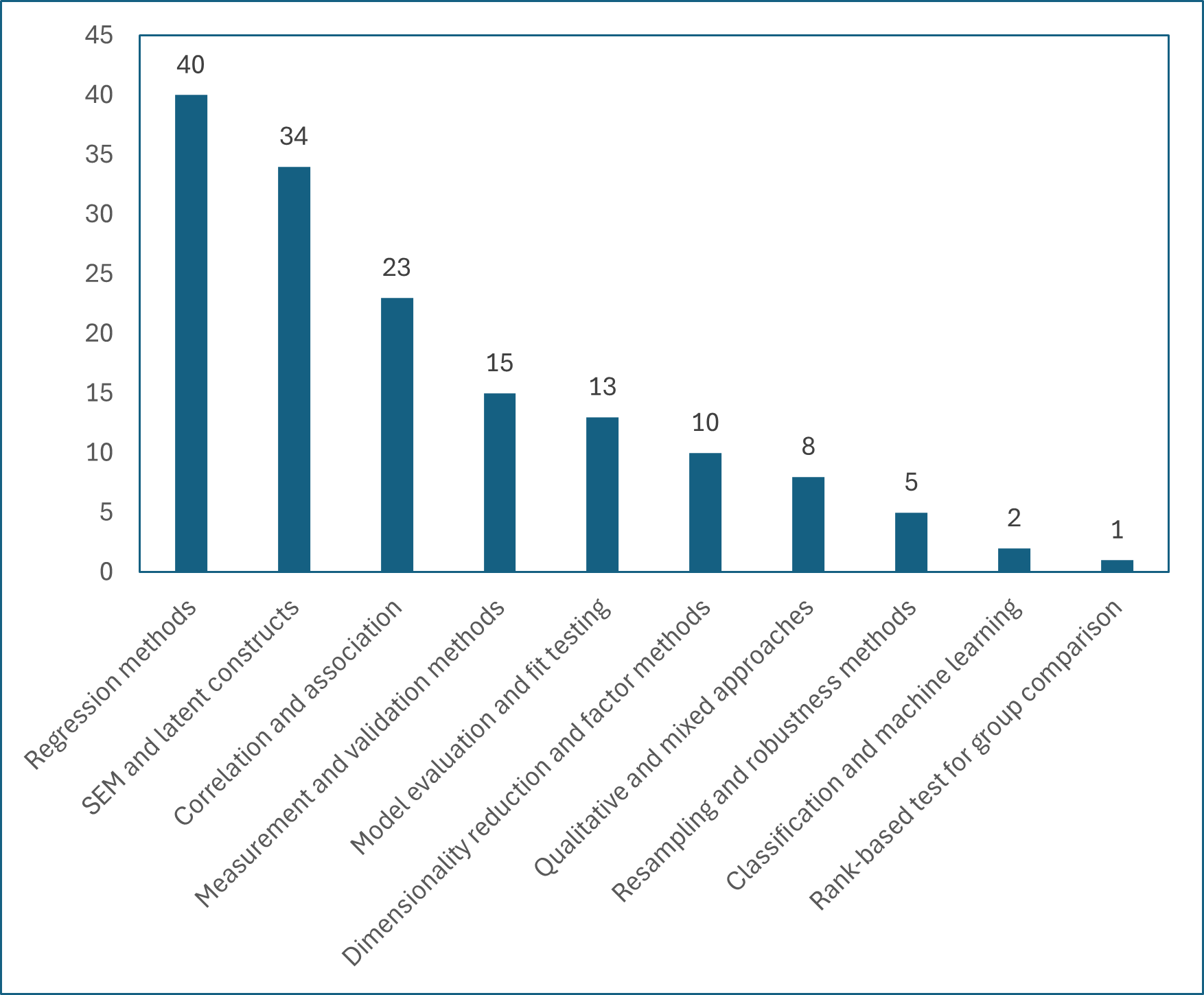

Supplement: Multimedia Appendix 7 [file humanfactors-v13-e86076-s007.png]
