# Supplementary material for: Predicting Electronic Health Record Usability: Scoping Review of Adoption Models, Metrics, and Future Directions
Source: JMIR Hum Factors. 2026 Apr 8;13:e86076. doi: 10.2196/86076 (PMC13060746; doi:10.2196/86076)
Supplement: Checklist 1 [file humanfactors-v13-e86076-s008.docx]

**Preferred Reporting Items for Systematic reviews and Meta-Analyses extension for Scoping Reviews (PRISMA-ScR) Checklist.**

This checklist follows the PRISMA-ScR) guidelines that corresponds to this study titled “Predicting Electronic Health Record (EHR) Usability: A Scoping Review of Models, Metrics, and Opportunities for Qualitative and Quantitative Analysis.” Each item is marked with the corresponding page number(s) where it appears in the manuscript, as well as a brief explanation.

Checklist adapted from: Tricco AC, et al. PRISMA Extension for Scoping Reviews (PRISMA-ScR): Checklist and Explanation. Ann Intern Med. 2018;169(7):467–473.

| Section | Item | PRISMA-ScR Checklist Item | Reported on Page(s) |
| --- | --- | --- | --- |
| TITLE | 1 | Identify the report as a scoping review. | 1 |
| ABSTRACT | 2 | Provide a structured summary including background, objectives, eligibility criteria, sources of evidence, charting methods, results, and conclusions. | 1 |
| INTRODUCTION | 3 | Describe the rationale for the review in the context of what is already known. | 2–3 |
|  | 4 | Provide an explicit statement of the objectives or questions the review addresses. | 3 |
| METHODS | 5 | Indicate whether a protocol exists; if registered, provide details (registration number, etc.). | 3 |
|  | 6 | Specify characteristics of sources of evidence used as eligibility criteria (e.g., years considered, language, publication status, study characteristics). | 5, Table 1 |
|  | 7 | Describe all information sources in the search (e.g., databases with dates of coverage). | 5-6 |
|  | 8 | Present the full electronic search strategy for at least one database, including any limits used. | 5–6 |
|  | 9 | State the process for selecting sources of evidence (screening, eligibility, inclusion). | 5-6 |
|  | 10 | Describe the methods of charting data from the included sources (e.g., forms used, independent extraction, confirmation). | 7 |
|  | 11 | List and define all variables (data items) for which data were sought and any assumptions made. | 5-7 |
|  | 12 | If critical appraisal of individual sources of evidence was performed, describe methods used and rationale. | Not applicable (no individual critical appraisal) |
|  | 13 | Describe methods for synthesizing and presenting the results. | 7-8 |
| RESULTS | 14 | Give numbers of sources screened, assessed for eligibility, and included, with reasons for exclusions, ideally using a flow diagram. | 9, Figure 1 |
|  | 15 | Present characteristics for each source of evidence for which data were charted. | 11–18, Table 2 |
|  | 16 | If critical appraisal was performed, present data on each source and summary of findings. | 7–18, column six called “Critique of Factors and Determinants” heading in Table 2 |
|  | 17 | Present results of individual sources of evidence as relevant to the review questions and objectives. | 7–18 |
|  | 18 | Summarize and present the charted data in relation to the review questions and objectives. | 19–22, Figures 2–9 |
| DISCUSSION | 19 | Summarize the main results (e.g., overview of concepts, themes, types of evidence). | 23–28 |
|  | 20 | Discuss limitations of the scoping review process. | 27 |
|  | 21 | Provide a general interpretation of the results with implications and/or next steps. | 27-28 |
| FUNDING | 22 | Describe sources of funding for the included sources of evidence and for the review itself. | 29, no conflict of interest and no funding |
